# A phase field finite element study and evaluation of sulfide stress cracking in DCB specimen testing

**DOI:** 10.1016/j.heliyon.2024.e39115

**Published:** 2024-10-10

**Authors:** Alok Negi, Mohamed Elkhodbia, Imad Barsoum, Akram AlFantazi

**Affiliations:** a Department of Mechanical and Nuclear Engineering, Khalifa University of Science and Technology, Abu Dhabi, 127788, United Arab Emirates; b Department of Engineering Mechanics, Royal Institute of Technology – KTH, Teknikringen 8, Stockholm, 100 44, Sweden; c Department of Chemical Engineering, Khalifa University of Science and Technology, Abu Dhabi, 127788, United Arab Emirates

**Keywords:** DCB, $K_{\mathrm{ISSC}}$, Stress-assisted diffusion, Phase field, Sulfide stress cracking, Finite element analysis

## Abstract

The challenges presented by sour environments rich in hydrogen sulfide (H_2_S) underscore the necessity for a comprehensive understanding of material behavior under such conditions. The cracking susceptibility of metals and alloys used for subsurface equipment in downhole oil and gas exploration operations is particularly concerning. The NACE Double Cantilever Beam (DCB) test has emerged as a widely used quality assurance tool in the petroleum industry, leveraging fracture mechanics principles to assess the environment-assisted cracking (EAC) resistance of metals and alloys. The DCB test evaluates the fracture toughness $K_{\mathrm{ISSC}}$ of materials in H_2_S-containing environments via assessment of the crack arrest, which serves as a vital parameter in structural integrity assessments to mitigate the risk of service-related failures from sulfide stress cracking (SSC). However, various studies suggest that different test parameters, such as arm displacement and initial notch, significantly influence $K_{\mathrm{ISSC}}$. This work presents a detailed numerical investigation on a comprehensive simulation of the DCB test, examining the effects of different test parameters on $K_{\mathrm{ISSC}}$ prediction. A coupled deformation-diffusion phase field framework is adopted to simulate SSC in DCB specimens arising from a complex interplay between material deformation, hydrogen diffusion, and fracture. The numerical results show good agreement with experimental results reported in the literature and provide deeper insights into the factors affecting crack growth and arrest in DCB testing.

## Highlights

• Numerical simulation of DCB test as per ANSI/NACE TM0177 Method D.
• Critical energy release rate as a function of hydrogen content.
• Influence of arm displacement on the measured value of *K*_ISSC_.
• Influence of initial notch on the measured value of *K*_ISSC_.

## 1 Introduction

The escalating demands of oil and gas production have propelled operational conditions in production wells to higher pressures and temperatures, particularly deep and ultra-deep wells. In these challenging conditions, metals such as high-strength carbon and low alloy steels, which are dominantly used in well completions, suffer from corrosion and environment-assisted cracking (EAC) issues [1], [2]. Particularly, sulfide stress cracking (SSC), a form of hydrogen embrittlement (HE), stands out as a formidable concern to the structural integrity of downhole tubulars and ancillary equipment in oilfield environments containing H_2_S coupled by the presence of high tensile stresses [3], [4], [5]. The impact of SSC can also extend beyond material degradation to the potential unforeseen or premature failures at relatively low-stress levels, posing a significant threat of substantial losses [1], [6]. Therefore, the resistance to cracking holds paramount significance in the selection process for materials in these demanding environments. The oil and gas industry currently employs the ANSI/NACE TM0177 [7], which delineates various laboratory testing methods designed to assess the performance of metals under the combined influence of tensile stresses and corrosion in aqueous environments containing H_2_S. These testing methods include standard tensile, bent beam, C-ring, and double cantilever beam (DCB) tests. Notably, the ANSI/NACE TM0177 Method D - Standard DCB test [7] has emerged as a widely adopted quality assurance measure for directly measuring the resistance of high-strength carbon and low alloy steels to SSC and stress-corrosion cracking (SCC).

**Figure 1:**
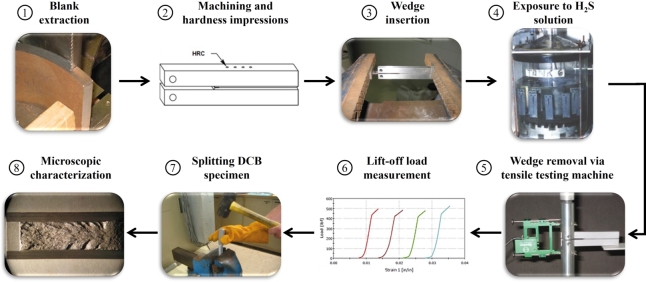
Different steps involved in DCB specimen testing [8], [9].

ANSI/NACE TM0177 method D is a crack arrest-type fracture mechanics test, which measures the resistance to SSC in terms of critical stress intensity factor (SIF) or fracture toughness ($K_{\mathrm{ISSC}}$) at or near ambient temperatures [10]. Fracture toughness is a generic term associated with the fracture mechanics methodology to assess material resistance to crack growth and finds application in structural integrity assessments, performance evaluation, damage tolerance design, and fitness-for-service evaluation to assess the risk of structural failure in service [11]. The DCB test serves a dual purpose: first, it aids in design and fitness-for-service evaluations through a reliable quantitative, consistent fracture mechanics-based criterion instead of other testing methods that provide a simple pass/fail outcome [12], [13]. Secondly, DCB specimens can be pre-stressed using a wedge before exposure to a corrosive H_2_S-containing environment, making it easier and cost-efficient than other fracture toughness test methods by facilitating various enclosed experimental fixtures, essential for H_2_S testing [14]. Fig. 1 presents a schematic depiction of the steps integral to conducting a DCB test [8] according to the method D in ANSI/NACE TM0177 standard [7].

Properly conducted, the DCB test has the potential to provide conclusive results in a single test using the DCB test, offering a distinct advantage over a series of fixed load tests required to establish a threshold. However, it is also acknowledged in the literature that $K_{\mathrm{ISSC}}$ is not an intrinsic material property but depends on the various test parameters for a particular sour medium [15], [16], [17], [18], [19]. For instance, test variables such as specimen geometry [16], arm displacement [17], [18], and crack starter geometry [19] have been shown to influence the measured value of $K_{\mathrm{ISSC}}$. In this work, a numerical study is conducted to study the effect of different test parameters on the measured value of the $K_{\mathrm{ISSC}}$ from the DCB test. During the DCB testing process, crack growth reflects a complex interplay among mechanical and chemical aspects, which modify the driving force for crack growth, thereby affecting cracking resistance. To this end, a coupled deformation-diffusion phase field approach is adopted to capture this interplay. Over the last two decades, smeared crack approaches have become a popular tool to address fracture-mechanics problems as an alternative to discrete approaches. Particularly, phase field (PF) formulations have become a promising computational tool to simulate fracture in multi-physics coupling problems such as chemo-mechanical [20], [21], [22], [23], [24], thermo-mechanical [25], [26], [27], electro-mechanical [28], [29], thermo-hydro-mechanical coupling [30], [31], [32], etc. PF models belong under variational approaches to fracture, where a sharp crack is geometrically regularized into a narrow band of intense damage using an internal length scale [33], [34], [35], [36]. PF models offer several advantages, such as eliminating the need for any ad hoc criteria to govern crack initiation and propagation, easy tracking of the evolving crack interface, no geometric constraints, and simplicity of implementation.

This study aims to simulate the DCB test according to the procedural steps defined in Method D of the ANSI/NACE TM0177 standard [7]. Sulfide stress cracking in DCB specimens due to exposure to H_2_S-containing environments is modeled using a coupled deformation-diffusion phase field framework that integrates stress-assisted hydrogen (H) diffusion, deformation, and fracture. To achieve precise and reliable results, the numerical methodology incorporates hydrogen permeation data and hydrogen-influenced fracture toughness data from the literature to accurately quantify boundary conditions related to hydrogen absorption and fracture toughness degradation in H_2_S environments. This involves establishing a correlation between these conditions and environmental factors, focusing on H_2_S content. Additionally, the study investigates the influence of test parameters, such as arm displacement and initial notch length, on the measured value of $K_{\mathrm{ISSC}}$. The manuscript is organized as follows: Section 2 briefly overviews the modeling framework underlying the coupled deformation-diffusion phase field approach. Section 3 presents the numerical results and discussions, highlighting key findings and their implications. Section 4 summarizes the conclusions drawn from this study.

## 2 Modeling framework

This section presents the governing equations of the coupled deformation-diffusion phase field framework assuming a small strain formulation.

### 2.1 Governing equations for mechanical problem with crack growth

Initially developed by Francfort and Marigo [37] to overcome limitations associated with the classical Griffith theory, a phase field model adopts a variational approach to fracture, which formulates brittle fracture as a minimization problem of total energy potential functional $\psi_{tot}$ associated with a cracked structure. Considering a solid body with a discontinuity *Γ*, this total potential energy functional $\psi_{tot}$ for a deformation-fracture problem includes a surface term associated with the energy dissipated in a crack along with elastic stored energy,

(1) $$\psi_{tot}(u,\Gamma )=\int_{\Omega }\psi_{e}(\epsilon (u))d\Omega +G_{c}\int_{\Gamma }d\Gamma$$

where $\psi_{e}$ is the elastic strain energy, $G_{c}$ is the critical energy release rate, and ***ϵ*** is an infinitesimal strain tensor,

(2) $$\epsilon (u)=\nabla_{s}u=\frac{1}{2}\left[\nabla u+\nabla^{T}u\right]$$

where $\nabla_{s}$ refers to the symmetric part of the strain tensor ***ϵ***. Bourdin et al. [38], [39] further reformulated this problem and proposed a regularized formulation of the $\psi_{tot}$ as,

(3) $$\psi_{tot}(u,\phi ,\nabla \phi )=\int_{\Omega }g(\phi )\psi_{e}(\epsilon (u))dV+G_{c}\int_{\Omega }\gamma (\phi ,\nabla \phi )dV$$

where *γ* is the crack density functional per unit volume, and g(ϕ) is a stress degradation function that captures material degradation using an auxiliary scalar phase field *ϕ* variable which characterizes the extent of damage at a material point, providing a smooth transition from an intact to a cracked region, such that ϕ=0 indicates an intact state and ϕ=1 represents a completely damaged state,

(4) $$g(\phi )=(1-\phi^{2})+k$$

Here, the parameter *k* is a numerical stability parameter chosen as small as possible to avoid singularity issues in numerical simulations. Restricting our analysis to linear elastic loading conditions, $\psi_{e}$ in the first part of the Eq. (3) is defined as,

(5) $$\psi_{e}(\epsilon (u))=\frac{1}{2}\epsilon :C:\epsilon$$

where **C** is the constitutive stiffness tensor for an intact material. Regarding the second part of Eq. (3), *γ* is a function of internal length scale *l*, which acts as a regularized parameter governing the width of the diffused crack and a scalar phase field ϕ∈[0,1] along with its spatial gradient ∇*ϕ*.

(6) $$\gamma (\phi ,\nabla \phi )=\frac{1}{2}\left(l\nabla \phi \cdot \nabla \phi +\frac{\phi^{2}}{l}\right)$$

After minimizing the functional $\psi_{tot}$ for displacement field **u** and phase field *ϕ* along with introducing a history variable $H_{v}$, the following coupled set of governing balance equations are obtained,

(7) ∇⋅σ=0 on Ω

(8) $$G_{c}\left(\frac{1}{l}\phi -l\nabla \phi \cdot \nabla \phi \right)-2(1-\phi )H_{v}=0\text{ on }\Omega$$

which are subjected to the following boundary conditions,

(9) $$\sigma \cdot n=t\text{, }u=\overset{¯}{u}\text{ and }\nabla \phi \cdot n=0\text{ on }d\Omega$$

where **n** is the outward unit normal to the boundary, $\overset{¯}{u}$ is the prescribed displacement, **t** is the prescribed traction, and ***σ*** is the Cauchy stress tensor. The equilibrium condition given in Eq. (7) ensures mechanical equilibrium, and the solution of the PDE given in Eq. (8) provides information on the spatial and temporal distribution of the phase field *ϕ* over time. To ensure the irreversibility of the phase field *ϕ* evolution without any crack healing, the history variable $H_{v}$ is expressed as,

(10) $$H_{v}=\max\left[\psi_{e}^{+}\right]$$

where $\psi_{e}^{+}$ is the positive part of the strain energy density. A commonly adopted energy split scheme proposed by Miehe [28] is adopted to prevent cracking in compression, such that the strain energy is additively decomposed as,(11)
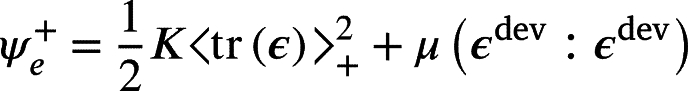
(12)
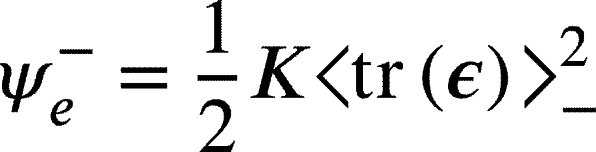
 where are *K* is the bulk modulus, *μ* is the shear modulus, $\epsilon^{\text{dev}}=\epsilon -\left(1/3\right)\mathrm{tr}\left(\epsilon \right)I$ is the deviatoric part of the strain tensor ***ϵ*** with **I** being a second order identity tensor, and the operator  denotes .

### 2.2 Governing equations for hydrogen transport

In the context of SSC, hydrogen transport refers to the diffusion of atomic hydrogen into the material on exposure to aqueous environments containing high content of H_2_S in the presence of high tensile stresses. Here, atomic hydrogen atoms refer to a product of the corrosion process that comprises chemical reactions between acidic aqueous components and the metal surface. The process generally begins with the generation of the hydrogen ions, which the following electrochemical reactions best describe the process where H_2_S dissolve and dissociate in an aqueous medium [40], [41].(13)
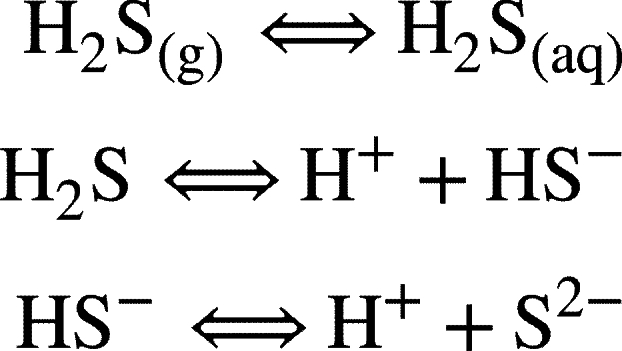
 At the same time, the metal atoms get oxidized, as in the case of steel, leaving electrons on the surface of the base metal, which is then absorbed by hydrogen ions to form free atomic hydrogen by proton reduction.(14)
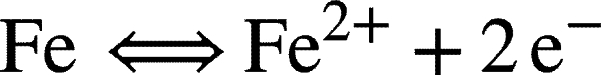
(15)
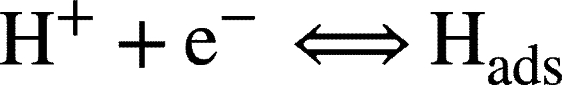


In most environments, most of the atomic hydrogen recombines on the metal surface to form hydrogen gas (H_2_), which harmlessly bubbles off the metal surface,(16)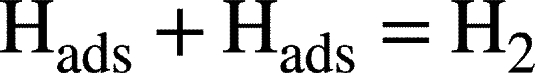 However, certain chemical species such as bisulfide ions (HS^−^) act as combination poisons, thereby retarding the kinetics of recombination and resulting in increased absorption of the atomic hydrogen into the lattice structure of metal [42]. The present numerical model adopts modified Fickian diffusion to describe hydrogen transport inside the metal after the chemisorption of atomic hydrogen, considering both concentration gradients and the gradient of hydrostatic stresses. The strong form of local mass balance for hydrogen transport reads,

(17) $$\frac{dC}{dt}+\nabla \cdot J=0\text{ on }\Omega$$

which is subjected to the following boundary conditions,

(18) $$J\cdot n=p\text{, }\text{ and }C=C_{b}\text{ on }d\Omega$$

where *p* and $C_{b}$ are the flux and concentration of hydrogen on the boundary. The hydrogen concentration *C* and $C_{b}$ are expressed in units of the units of weight part per million (wppm) throughout this work. The hydrogen permeation flux **J** is related to the gradient of the chemical potential ∇*μ* through a linear Onsager relationship based on Einstein's equation of diffusion,

(19) $$J=-\frac{DC}{RT}\nabla \mu$$

where *D* is an hydrogen diffusivity, *R* is a gas constant (R=8.314 $\mathrm{Jmo}l^{-1}K^{-1}$), *T* is the absolute temperature (*T*= 298.15 K), and *μ* is the chemical potential that drives hydrogen diffusion. During hydrogen permeation studies in H_2_S-saturated aqueous test solutions, it is observed that the surface of the testing specimens in contact absorbs a significant amount of hydrogen, gradually attaining an equilibrium state leading to a uniform subsurface hydrogen concentration under steady-state conditions [43], [44], [45]. A constant Dirichlet-type boundary condition is adopted in this work, where a constant hydrogen concentration $C=C_{b}$ is imposed on the boundaries of the DCB testing specimen to reflect uniform subsurface hydrogen on the boundaries under steady-state conditions.

### 2.3 Fracture toughness degradation

The transport of hydrogen and its distribution inside any material is not homogeneous. Instead, the hydrogen atoms get arrested at microstructural imperfections (e.g., carbides or grain boundaries) called trap sites, leading to localized segregation, which induces the degradation of material properties, notably fracture toughness [46], [47]. In this work, the influence of hydrogen diffusion on the fracture toughness is captured through the following relation [48],

(20) $$G_{c}=q(C)G_{c}(0)$$

where $G_{c}(0)$ is the critical energy release rate of the material without the influence of hydrogen and q(C) is a degradation function that relates $G_{c}$ with hydrogen concentration *C*,

(21) $$q(C)=\left[\frac{G_{c}^{min}}{G_{c}(0)}+\left(1-\frac{G_{c}^{min}}{G_{c}(0)}\right)\text{exp}\left(-\alpha C\right)\right]$$

where *α* is a fitting parameter, and $G_{\text{min}}$ refers a saturation magnitude which is the lowest value of $G_{c}$ for a particular material under consideration in sour environment. Note that the critical energy release rate $G_{c}$ can be calculated from the fracture toughness $K_{Ic}$ using the standard conversion for a linearly elastic material.

### 2.4 Stress-driven hydrogen diffusion coupled with phase-field fracture

The total free energy density for the coupled deformation-diffuse-fracture problem, which satisfies the energy dissipation condition, is defined following the work by Martínez-Pañeda et al. [20],

(22) $$\psi (u,\phi ,\nabla \phi ,C)=\underset{\psi^{b}}{\underset{︸}{g(\phi )\psi_{e}(\epsilon )-K{\overset{¯}{V}}_{H}\left(C-C^{0}\right)\text{tr}(\epsilon )}}+\underset{\psi^{s}}{\underset{︸}{G_{c}(C)\gamma (\phi ,\nabla \phi )}}+\underset{\psi^{c}}{\underset{︸}{\mu^{0}C+RTN\left(\theta_{L}\ln\theta_{L}+\left(1-\theta_{L}\right)\ln\left(1-\theta_{L}\right)\right)}}$$

where $\psi^{b}$, $\psi^{s}$, and $\psi^{c}$ denote chemo-elastic energy, crack surface energy, and chemical-free energies stored in the bulk. The symbol $C^{0}$ is the reference lattice hydrogen concentration, tr(ϵ) is the trace of a second order tensor ***ϵ***, $\mu^{0}$ is the reference chemical potential, ${\overset{¯}{V}}_{H}$ is the partial molar volume of hydrogen, $\theta_{L}=C/N$ is the lattice hydrogen occupancy and *N* is the number of lattice sites. Based on the total chemo-mechanical free energy given in (22), constitutive prescriptions can be derived in a thermodynamically consistent manner [20]. For example, the Cauchy stress tensor is constructed as,

(23) $$\sigma =\frac{\partial \psi }{\partial \epsilon }=g(\phi )\sigma_{0}-K{\overset{¯}{V}}_{H}\left(C-C^{0}\right)I$$

where $\sigma_{0}=C:\epsilon$ is the stress tensor associated with the undamaged state. The second term in Eq. (23) is neglected considering its negligible contribution in case of hydrogen embrittlement problems [49], [20]. Similarly, the chemical potential *μ* can be derived from the chemo-mechanical free energy as,

(24) $$\mu =\frac{\partial \psi }{\partial C}=\mu^{0}+RT\ln\left(\frac{\theta_{L}}{1-\theta_{L}}\right)-{\overset{¯}{V}}_{H}\sigma_{H}+G_{c}^{\prime }(C)\gamma (\phi ,\nabla \phi )$$

where $\sigma_{H}$ refers to hydrostatic (mean) stress $\sigma_{H}=\text{tr}(\sigma )/3=(\sigma_{11}+\sigma_{22}+\sigma_{33})/3$. The last part of Eq. (24) is neglected in this work that requires appropriate chemical boundary conditions in the presence of a propagating crack [20]. Further, assuming low site occupancy $\theta_{L}<<1$ such that $\theta_{L}/(1-\theta_{L})\approx \theta_{L}$ and ∇N=0 (constant hydrogen trapping density), the hydrogen diffusion flux **J** takes the following form,

(25) $$J=-D\nabla C+\frac{DC}{RT}{\overset{¯}{V}}_{H}\nabla \sigma_{H}$$

Refer to Appendix A for the finite element implementation details of the coupled modeling framework.

## 3 Numerical simulation

This section provides a detailed discussion of the virtual DCB testing for C110 low alloy steel to predict $K_{\mathrm{ISSC}}$ in low-pH H_2_S-saturated aqueous environments. C110 is a common high-strength steel with extensive application in the petroleum industry, particularly in deep sour condensate wells. Table 1 lists the material parameters used for C110 steel. Note that the wedge is constructed from the same material or class as the DCB specimen [7]. The DCB test is divided into three distinct steps: loading the wedge to induce a constant displacement, exposure to the aqueous test solution, and finally, the measurement of liftoff load. In this work, the DCB and wedge geometry are modeled as a 2D plain strain problem. The transient crack growth (i.e., sulfide stress cracking) in DCB specimen under exposure to the aqueous test solution (NACE solution A - 100% H_2_S) is captured using the coupled deformation-diffusion phase field framework discussed in Section 2. The data provided by different experimental investigations are used to validate the numerical results. All the numerical simulations were conducted using FE Software ABAQUS CAE.

**Table 1:** Material parameters used for the OCTG-grade C110 steel.

Parameters	Value	Units	Ref.
Elastic modulus *E*	207	GPa	[7]
Yield strength *σ*_*YS*_	850	MPa	[50]
Poisson's ratio *ν*	0.3	-	[7]
Fracture toughness *K*_*Ic*_	117.99	MPa $\sqrt{m}$	[50]

### 3.1 Determination of $K_{\mathrm{ISSC}}$

**Figure 2:**
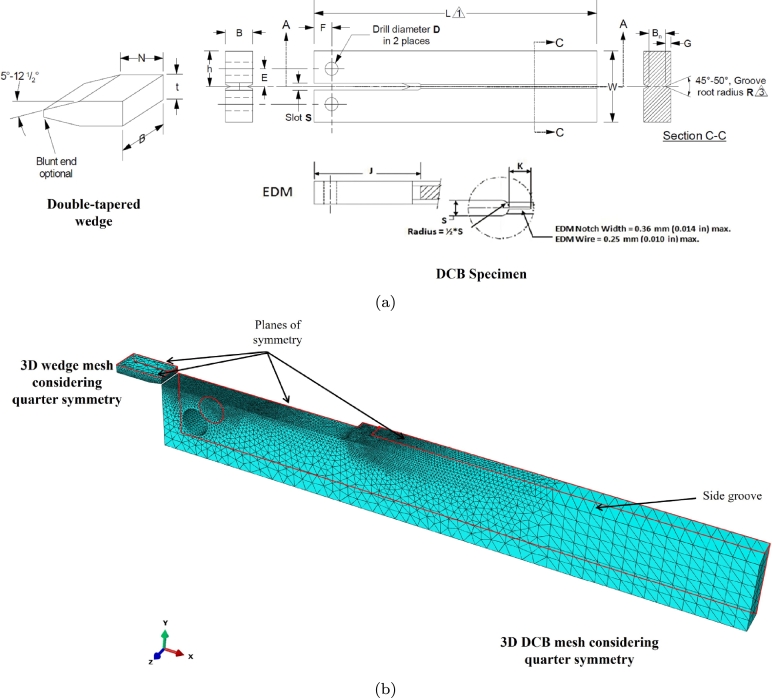
(a) Geometric description of DCB specimen and double-tapered wedge provided in ANSI/NACE TM0177 [7] (top), and (b) Finite element mesh details of (a) 3D quarter-DCB specimen containing 220067 quadratic tetrahedral elements (C3D10), and quarter double-tapered wedge containing 13758 quadratic tetrahedral elements (C3D10).

The determination of $K_{\mathrm{ISSC}}$ follows the concept of decaying K loading and crack arrest in a H_2_S containing environment. The $K_{\mathrm{ISSC}}$ for a flat 2D DCB specimen without side grooves is computed based on the original work of Heady [52],

(26) $$K_{\mathrm{ISSC}}=\frac{Pa\left(2\sqrt{3}+2.38\left(\frac{h}{a}\right)\right)}{Bh^{\frac{3}{2}}}$$

where *P* is the measured final lift-off load, *a* is the final crack length identified by opening the DCB specimen mechanically, *h* is the half specimen height, and *B* is the specimen thickness. After the exposure, the wedge should be removed from the DCB specimen at a minimum displacement rate of 0.5 mm/min in the tensile testing machine setup [7]. In this process, the lift-off load *P* is measured, representing the equilibrium wedge load, measured from the abrupt change in slope of the “Lift-off” curve or load-displacement curve when removing the wedge from the DCB specimen using a tensile testing machine. A modification of Eq. (26) is suggested in Heady [52] for DCB specimen with side grooves, which is currently adopted in ANSI/NACE TM0177 Method D [7],

(27) $$K_{\mathrm{ISSC}}=\frac{Pa\left(2\sqrt{3}+2.38\left(\frac{h}{a}\right)\right){\left(\frac{B}{B_{n}}\right)}^{\frac{1}{\sqrt{3}}}}{Bh^{\frac{3}{2}}}$$

where $B_{n}$ represents the web thickness. Fig. 2a presents the complete geometric details of the DCB specimen and double-tapered wedge, as outlined in ANSI/NACE TM0177 [7]. Note that the factor $B/B_{n}$ in Eq. (27) captures the reduction in the width of the crack plane to facilitate stable crack growth. Following the work of Heady [52], an ASTM plane-strain criterion was introduced as a mandatory requirement to validate the test, which is also included in the initial revision of the ANSI/NACE TM0177 standard,

(28) $$B\geq 2.5\times \left(\frac{K_{\mathrm{ISSC}}}{\sigma_{YS}}\right)$$

where $\sigma_{YS}$ is the yield strength.

### 3.2 Choice of thickness *B*

**Figure 3:**
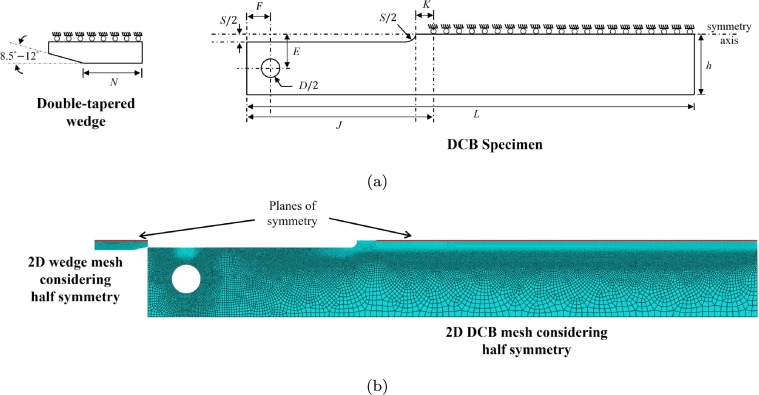
(a) Schematic description of a 2D DCB specimen and double-tapered wedge considering half symmetry, and (b) Finite element mesh details of 2D DCB specimen containing 299175 biquadratic plain strain quadrilateral elements (CPE8R) and double-tapered wedge containing 5575 biquadratic plain strain quadrilateral elements (CPE8R).

In this section, multiple finite element simulations are performed for wedge-loading and lift-off simulations to identify a suitable choice for thickness *B* for the DCB specimen. The wedge-loading is performed by inserting a double-tapered wedge into the gap between the arms of the test specimen, establishing an initial applied SIF ($K_{\text{Iapplied}}$). Meanwhile, the initial lift-off load is measured by simulating a tensile testing setup considering room conditions. Fig. 2a and Fig. 3a present the schematic details of the DCB specimen with an electro-discharge machine (EDM) notch and a double-tapered wedge, for the 3D and 2D problem [7]. Fig. 2b and Fig. 3b show the finite element mesh details of the DCB specimen and double-tapered wedge for the respective 3D and 2D simulations, where a relatively fine mesh is adopted in the crack-tip region and contact region. Only a quarter of the DCB specimen is modeled for the 3D problem (considering quarter symmetry) by imposing symmetry conditions on the planes (XY and XZ) for both the DCB specimen and the double-tapered wedge. Meanwhile, for the 2D problem, only half of the DCB specimen is modeled considering half symmetry. Table 2 lists the complete dimensions for the DCB specimen and a double-tapered wedge. The geometry dimensions used here correspond to ANSI/NACE TM0177–2016 Method D [7].

**Table 2:** Geometric dimensions of the standard DCB specimen with an EDM notch as per the NACE TM0177-2005 Method D [51] and ANSI/NACE TM0177–2016 Method D [7].

*L*	*B*	*B*_*n*_	*h*	*S*	*E* = *F*	*J*	*D*	*K*	*N*	*a*_*i*_[=*J* − *F*]	Ref.
101.60	9.53	5.72	12.70	2.39	6.35	41.3	4.85	3.17	6.35	34.95	[51]
101.60	9.53	5.72	12.70	2.39	6.35	38.10	4.85	3.18	6.35	31.75	[7]

**Figure 4:**
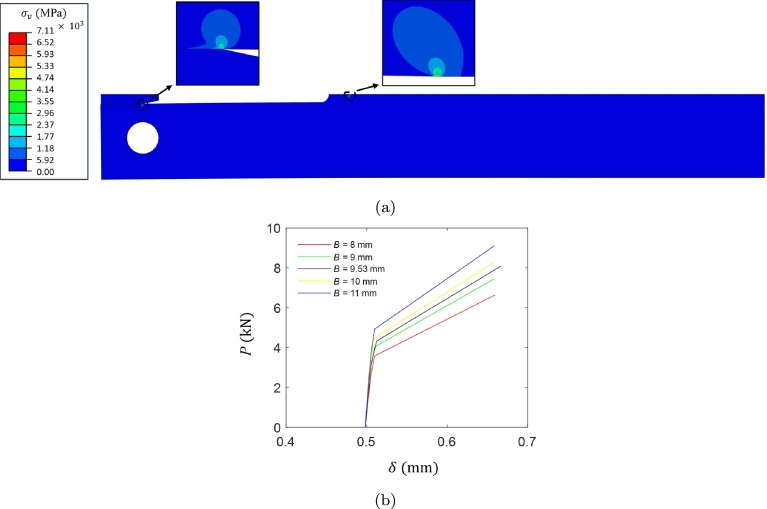
(a) von Mises stress *σ*_*v*_ distribution at the end of wedge loading, and (b) measured lift-off curves for a target arm displacement *δ* of 0.51 mm for different thickness *B* values.

Fig. 4a presents the von Mises stress distribution inside the DCB and wedge at the end of wedge insertion. A target arm displacement of 0.51 mm (i.e., δ/2=0.255 mm considering wedge symmetry) is selected for the wedge loading. The simulation of the DCB specimen opening involves a downward displacement from the top of the wedge through wedge insertion until the desired arm displacement is achieved. The contact between the DCB arms and the wedge is simulated as normal with hard contact and tangential behavior with a friction coefficient of 0.4. Upon contact with the specimen, the specimen undergoes a loading dominated by an opening mode (mode I). The second part of this numerical analysis involves measurement of the lift-off load and computation of $K_{\text{Iapplied}}$ using Eq. (26). A displacement rate of 0.24 mm/min is maintained in the lift-off simulations. Fig. 4b presents the lift-off curves of the 2D problem, where the steep portion of the curve represents the removal of compression on the wedge and the upper, shallower part indicates the compliance of the DCB specimen itself. Note that the lift-off load is not the actual load imparted by the wedge but the force necessary to remove the compression of the wedge. Table 3 compares the $K_{\text{Iapplied}}$ values obtained through 3D and 2D finite element simulations for various thicknesses of the DCB specimen. The results exhibit an insignificant difference between $K_{\mathrm{ISSC}}$ values obtained using 2D and 3D finite element simulations. Satisfying the ASTM plane-strain criterion in Eq. (28) (i.e., minimum necessary thickness), a thickness of B=9.53 mm is a suitable choice for the 2D plain strain problem.

**Table 3:** *K*_Iapplied_ for different thickness *B* values.

	$a_{i}$ (mm)	*B* (mm)	*P* (N)	$K_{\text{Iapplied}}$
3D FEM	31.75	9.53	3230.00	44.5845

2D FEM	31.75	8	3581.75	43.8614
		9	4028.66	43.8526
		9.53	4323.66	44.4463
		10	4475.38	43.8437
		11	4922.02	43.8357

### 3.3 Choice of hydrogen diffusion parameters

A fundamental component of this investigation involves the precise selection of hydrogen diffusion parameters and subsurface hydrogen boundary conditions. In this work, these parameters are derived from comprehensive hydrogen permeation studies from the literature. Hydrogen permeation testing is a widely accepted methodology for empirically deriving parameters associated with diffusion and trapping phenomena in metals and alloys through permeation transient fitting techniques [53], [54], [55]. Here, the steady-state subsurface hydrogen concentration ($C_{0}$) and diffusion coefficient ($D_{app}$) obtained from hydrogen permeation tests serve as input to the hydrogen transport model to reflect the accurate selection of boundary conditions and hydrogen diffusion due to acidic aqueous test solutions containing H_2_S. A steady-state subsurface hydrogen concentration ($C_{0}$) reflects a constant hydrogen flux during a steady state. The $C_{0}$ value is derived from hydrogen permeation experiments and approximates $C_{b}$ in our numerical simulations, facilitating an accurate representation of boundary conditions.

**Figure 5:**
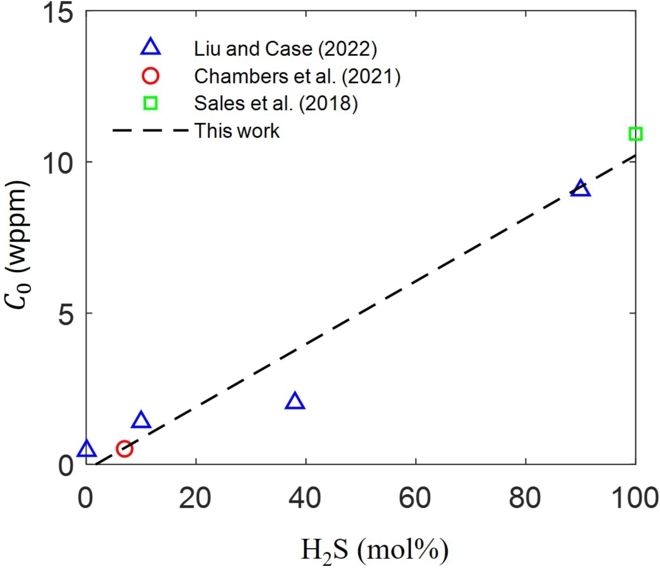
Sub-surface hydrogen concentration *C*_0_ as a function of H_2_S content in aqueous test solutions in mol % [43], [44], [45].

Fig. 5 presents sub-surface hydrogen concentrations $C_{0}$ obtained through permeation transient fitting for API C110 as a function of H_2_S concentration (mol%). Chambers et al. [57] conducted hydrogen permeation tests on API C110, where $C_{0}$ varies from 0.47 to 0.67 wppm, and $D_{app}$ from $1.24\times 10^{-6}$ $cm^{2}/\sec$ to $1.57\times 10^{-6}$ $cm^{2}/\sec$ for different samples. In Liu and Case [44], hydrogen permeation tests for different H_2_S concentrations ranging from 0 mol% to 90 mol% in the testing solutions. This work adopts a linear approximation of the $C_{0}$ for $C_{b}$, shown by a blue curve in Fig. 5. The hydrogen diffusivity $D=D_{app}=1.3\times 10^{-6}$ $cm^{2}/\sec$ is adopted [58], [59]. The partial molar volume of hydrogen, i.e., ${\overset{¯}{V}}_{H}$, is assumed to be 2000 $mm^{3}/\mathrm{mol}$, a common value in studies related to hydrogen transport in iron-based materials. The temperature (*T*) is set to 298.15 K, adhering to room temperature conditions per the ANSI/NACE TM0177 standard.

### 3.4 Hydrogen-induced fracture toughness degradation

**Figure 6:**
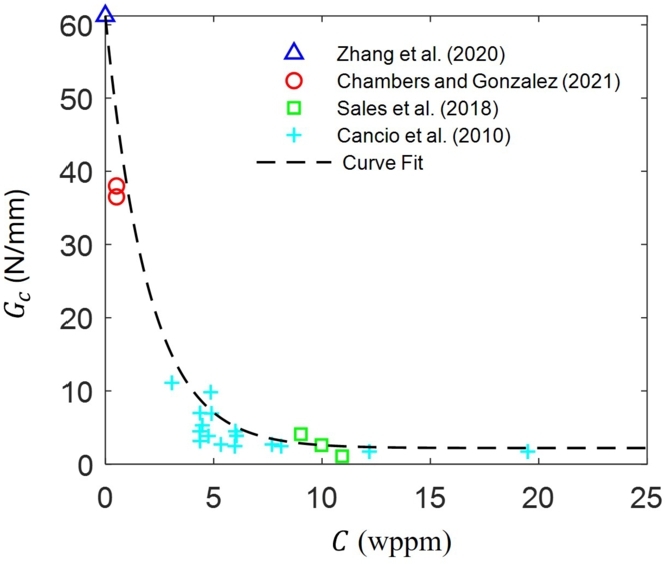
Critical energy release rate *G*_*c*_ as a function of hydrogen concentration *C*[45], [56], [50], [43].

Apart from the hydrogen permeation studies, multiple experimental studies can be found in the literature on the variation of $K_{\mathrm{ISSC}}$ for different aqueous test solutions. Typically, ANSI/NACE TM0177 specifies different H_2_S-saturated aqueous test solutions, such as A, B, C, and D, with different levels of severity, with NACE A solution and NACE solution D solution representative of severely sour and mild sour conditions. Fig. 6 shows the variation of $G_{c}$ as a function of hydrogen concentration *C* adopted in this work, with α=0.5 and $G_{c}^{\text{min}}$ assumed to be 2.2 N/mm. The curve fit is based on works in the literature involving experimental $K_{\mathrm{ISSC}}$ measurements and hydrogen permeation tests [45], [56], [50], [43] on C110 steel and other high strength steels, where $G_{c}$ is calculated from the $K_{\mathrm{ISSC}}$ using the standard conversion for a linearly elastic material under plain strain conditions, i.e., $K_{Ic}=\sqrt{EG_{c}/\left(1-\nu^{2}\right)}$. The $G_{c}(0)=61.21$ N/mm in Fig. 6 corresponds to the fracture toughness measurement in Zhang et al. [50] for C110 steel at normal temperature and pressure, without the influence of H_2_S.

### 3.5 Virtual DCB test

The numerical analysis is divided into three distinct steps. The first step refers to a wedge insertion into the gap between the arms of DCB specimens, establishing an initial applied SIF ($K_{\text{Iapplied}}$) through a slight displacement of the arms, referred to as arm displacement. The second step is a coupled deformation-diffusion modeling to simulate crack growth under the effect of H_2_S exposure and a constant displacement induced by the wedge. The last step is the measurement of liftoff load from the lift-off curve and computation of $K_{\mathrm{ISSC}}$ using. Refer to Section 3.2 for the numerical implementation details for the first and last step. Note that all three steps adopt the same FE mesh for the DCB. Fig. 3b presents the finite element mesh details of the DCB specimen and double-tapered wedge adopted for these simulations.

#### 3.5.1 Exposure to aqueous test solution

**Figure 7:**
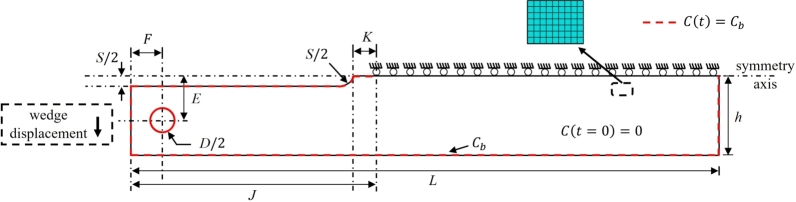
Geometry and boundary conditions for the coupled deformation-diffusion phase field problem.

Following the wedge insertion for a suitable arm displacement, the DCB-wedge assembly is exposed to an aqueous test solution, where the crack growth reflects the decrease in the internal elastic deformation energy in the DCB, with the H_2_S containing environment being the primary driving force behind crack growth [60]. In this part, coupled deformation-diffusion phase field finite element simulations are conducted to simulate crack growth within the DCB specimen exposed to aqueous test solution containing H_2_S. The boundary conditions associated with this problem are shown in Fig. 7, where a uniform hydrogen concentration is imposed on the boundaries of the DCB specimen (refer to dashed red line on the DCB boundary in Fig. 7). The magnitude of hydrogen concentration on the boundaries $C_{b}$ is derived from the linear trend shown in Fig. 5, corresponding to NACE solution A (100% H_2_S exposure). Apart from the hydrogen boundary conditions, a constant target arm displacement *δ* is maintained during this step induced by wedge insertion. The initial crack in DCB specimen is defined geometrically by not enforcing the symmetry ($u_{y}=$ 0) condition on the crack surface.

**Figure 8:**
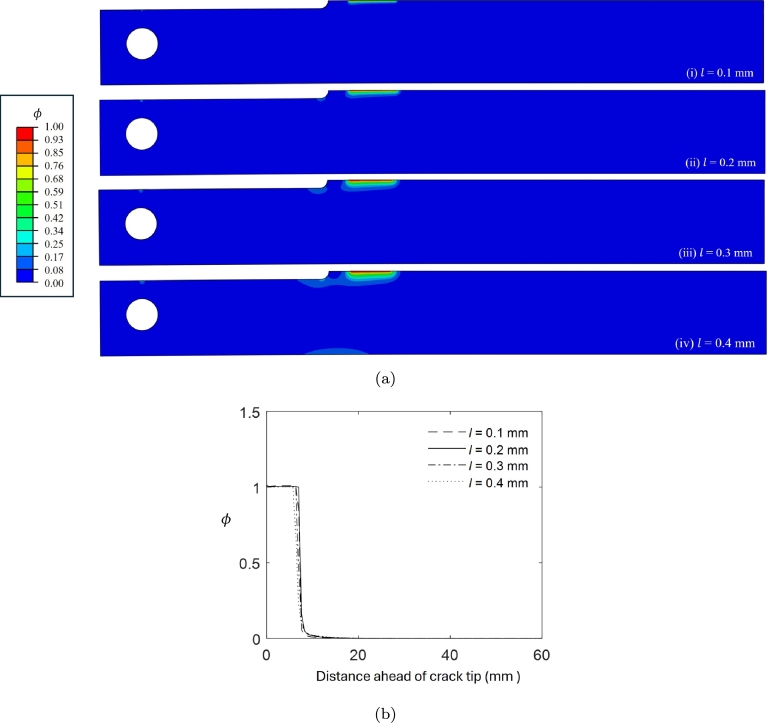
(a) Crack path at time *t* = 138 hours for (i) *l* = 0.1 mm, (ii) *l* = 0.2 mm, (iii) *l* = 0.3 mm, and (iv) *l* = 0.4 mm; (b) Variation of crack phase field *ϕ* ahead of the crack tip in the DCB.

Concerning the phase field *ϕ*, an internal length scale of *l*= 0.2 mm is adopted for all the numerical simulations, ensuring that the characteristic element size is $p_{e}<=(l/10)$ in the regions where phase field growth is expected. To validate this choice of *l*, we performed different coupled deformation-diffusion phase field simulations considering four different internal length scales (*l* = 0.1, 0.2, 0.3, and 0.4 mm) under identical conditions ($a_{i}$ = 31.75 mm, *δ*= 0.51 mm, and H_2_S exposure). Fig. 8a shows that the crack profiles at *t* = 138 hours are nearly identical across all cases, displaying a smooth transition of the phase field from ϕ=1 to ϕ=0 in the uncracked regions, with only a difference in the width of the damage zone, which increases for larger length scales. This observation highlights the role of length scale parameter in controlling the bandwidth of phase field distribution in the DCB, where larger length scales result in broader damage zones. Fig. 8b illustrates the variation of *ϕ* ahead of the crack tip, revealing negligible differences in crack growth for different length scales, thereby justifying the selection of l=0.2 mm for the simulations.

**Figure 9:**
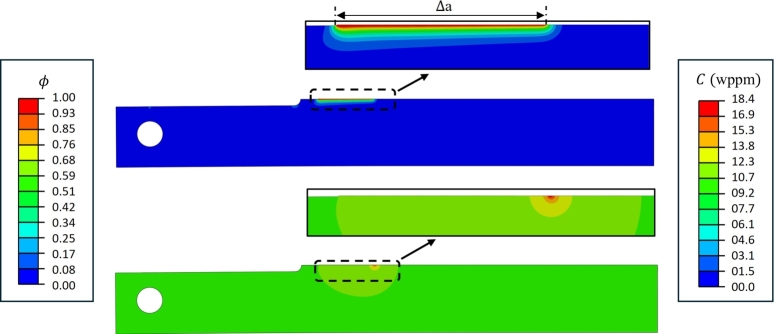
Contour plots for distributions of crack phase field *ϕ* (top), and hydrogen concentration *C* (bottom) at *t* = 336 hours.

Fig. 9 shows the contour plots for the distributions of the phase field and hydrogen concentration at t=336 hrs. The symbol Δa indicates the crack growth over the specified time of H_2_S exposure, such that phase field variable ϕ≈1. As shown in Fig. 9, hydrogen infiltrates the fracture process zone ahead of the crack tip, where the hydrostatic stress is highest, ultimately leading to crack growth along the uncracked ligament of DCB due to a local reduction in the critical energy release rate $G_{c}$. The crack growth stops when $K_{\text{Iapplied}}$ reduces to a critical $K_{\mathrm{ISSC}}$ value of material in the specified environment. The numerical results here correspond to the DCB test simulation considering a target arm displacement of 0.51 mm, where the distribution of crack phase field *ϕ* indicates a final crack length ($a=a_{i}+\Delta a$) of 41.90 mm for NACE solution A, representative of severe sour conditions.

#### 3.5.2 Effect of arm displacement

**Figure 10:**
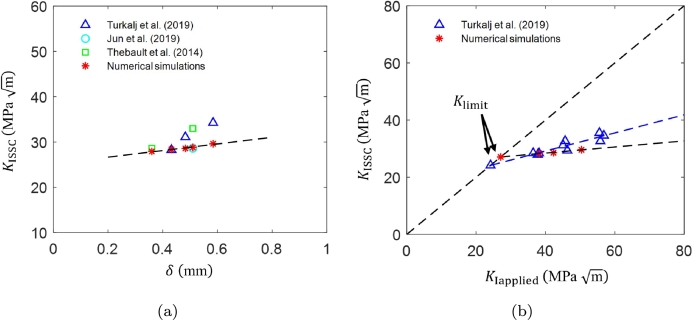
Numerically and experimentally determined (a) *K*_ISSC_ as a function of arm displacement [16], [18], [58], and (b) *K*_limit_ and *K*_ISSC_ measurements as a function of *K*_Iapplied_[18].

Although the DCB test has been standardized in the NACE standard TM0177 Method D, it is also increasingly acknowledged in the literature that different test variables can affect the test results. For instance, several works have highlighted the unexpected effect of arm displacement *δ* through the $K_{\text{Iapplied}}$ on the measured value of $K_{\mathrm{ISSC}}$ [61]. In this section, the effect of the arm displacement on $K_{\mathrm{ISSC}}$ is investigated, and a $K_{\mathrm{limit}}$ is identified for the DCB test. $K_{\mathrm{limit}}$ refers to the lowest $K_{\mathrm{ISSC}}$ value accessible by the DCB test for a material in a specific sour environment. Fig. 10a shows the variation of $K_{\mathrm{ISSC}}$ for different arm displacements using numerical simulations. The results show a linear variation in $K_{\mathrm{ISSC}}$ with increasing arm displacement, aligning with experimental trends. Both experimental and numerical results indicate that $K_{\mathrm{ISSC}}$ increases linearly with arm displacement, underscoring the significant influence of arm displacement on $K_{\mathrm{ISSC}}$.

**Figure 11:**
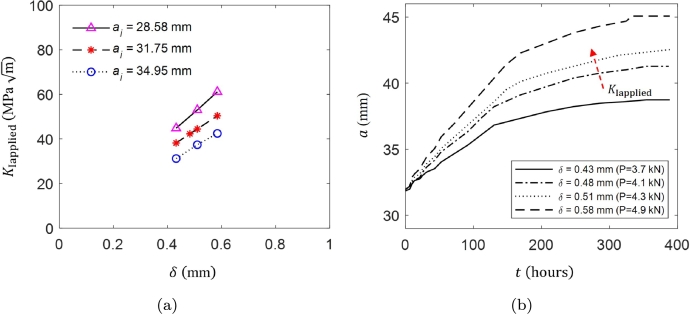
(a) *K*_Iapplied_ as a function of applied target arm displacement *δ*, and (b) Numerically computed crack growth response for different target arm displacements.

To better understand the effect of arm displacement, we examine its impact on $K_{\text{Iapplied}}$ and the crack growth response over time. Fig. 11a shows that $K_{\text{Iapplied}}$ increases proportionally with both arm displacement and initial crack length. Fig. 11b presents the numerically computed crack growth response for DCB specimens ($a_{i}=$ 31.75 mm) under different arm displacements. The crack growth increases rapidly during the initial steps for all the cases but slows down over time, likely due to the constant applied load. Note that a larger arm displacement (i.e., higher $K_{\text{Iapplied}}$) will induce more significant stress concentration at the crack tip, leading to elevated local hydrogen concentration and an accelerated crack growth rate driven by the concentration gradient. Ideally, the DCB specimen with a higher $K_{\text{Iapplied}}$ should propagate more distance to achieve the same $K_{\mathrm{ISSC}}$ compared to a specimen with a smaller $K_{\text{Iapplied}}$ and vice versa. However, Fig. 11b shows that the crack growth decelerates with increasing arm displacement resulting in increased lift-off loads. This explains a linear increase in $K_{\mathrm{ISSC}}$ with increasing arm displacement in Fig. 10a.

As discussed, for a given test duration, an initial $K_{\text{Iapplied}}$ directly affects the measured $K_{\mathrm{ISSC}}$ measurement. For example, a given initial $K_{\text{Iapplied}}$ provides a certain $K_{\mathrm{ISSC}}$, which is not a true indicator of crack arrest. As a result, the concept of $K_{\mathrm{limit}}$ becomes important because it defines a threshold below which SSC is unlikely to propagate in the components with defects that experience an in-service $K_{\text{Iapplied}}$ below the $K_{\mathrm{limit}}$. Herein, three specific arm displacements are considered for $K_{\mathrm{limit}}$ measurement, i.e., *δ*= 0.43 mm, 0.48 mm, and 0.58 mm, similar to the experimental program in Turkaji [18], where DCB tests were conducted on API 5CT-C110 grade DCB specimens ($a_{i}=$ 31.75 mm) for different arm displacements in NACE A solution (pH = 2.7). Fig. 10b presents the numerical and experimental $K_{\mathrm{ISSC}}$ measurements for three targeted arm displacements. The $K_{\mathrm{limit}}$ value is determined by extrapolating the slope to lower $K_{\text{Iapplied}}$ leads to an intersection with a 45^∘^ line. In Turkaji [18], a $K_{\mathrm{limit}}$ value was measured as 24.15 MPa $\sqrt{m}$ in NACE A solution. In comparison, the numerical results provide reasonable predictions with a $K_{\mathrm{limit}}$ of 27.08 MPa $\sqrt{m}$.

#### 3.5.3 Effect of initial notch

**Figure 12:**
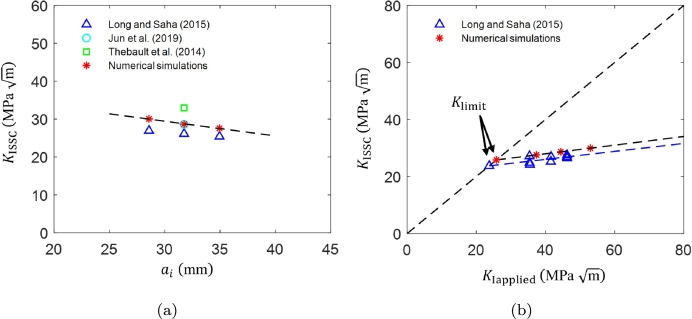
Numerically and experimental determined (a) *K*_ISSC_ as a function of initial crack length *a*_*i*_[16], [17], [58], and (b) *K*_limit_ and *K*_ISSC_ measurements as a function of *K*_Iapplied_[17].

In addition to arm displacement, initial notch or initial crack length affects the $K_{\text{Iapplied}}$ and ultimately influences the $K_{\mathrm{ISSC}}$ and $K_{\mathrm{limit}}$ measurement. Previous studies have shown that varying the initial crack length can lead to different stress intensity factors and thereby affect crack propagation behavior under sour environmental conditions [17]. In this section, the effect of the initial notch is investigated considering different initial crack lengths, $a_{i}=$ 28.58 mm, 31.75 mm, and 34.95 mm, similar to the work in Long and Saha [17] where DCB tests are performed in NACE solution A in accordance to ANSI/NACE TM0177 standard. Fig. 12a shows numerically computed and experimental $K_{\mathrm{ISSC}}$ values as a function of initial crack length $a_{i}$ for the arm displacement *δ*= 0.51 mm. The numerical results align well with the experimental trends, demonstrating that a shorter initial crack length results in higher values of $K_{\text{Iapplied}}$ and $K_{\mathrm{ISSC}}$.

Moreover, the results reveal a linear relationship between $K_{\mathrm{Iapplied}}$ and $K_{\mathrm{ISSC}}$, emphasizing the need to determine $K_{\mathrm{limit}}$, in line with prior experimental studies. Fig. 12b shows the numerical and experimental $K_{\mathrm{limit}}$ and $K_{\mathrm{ISSC}}$ measurements for three different initial crack lengths. A $K_{\mathrm{limit}}$ value of 25.82 MPa $\sqrt{m}$ is measured compared to the experimental prediction of 23.74 MPa $\sqrt{m}$ [17]. Similar to the role of target arm displacement discussed in Section 3.5.2, initial crack length also affects the final $K_{\mathrm{ISSC}}$ value, emphasizing the importance of accounting for initial crack length when assessing the fracture toughness of materials in sour environments. Notably the initial crack length dictates the $K_{\text{Iapplied}}$ (see Fig. 11a), such that shorter initial cracks induce higher $K_{\text{Iapplied}}$ compared to longer initial cracks. As a result, short initial crack length results higher $K_{\mathrm{ISSC}}$ compared to longer initial crack.

## 4 Conclusions

The DCB test, outlined in ANSI/NACE TM0177 standard Method D, is widely adopted in the oil and gas industry as a quality assurance test for assessing the susceptibility of metals and alloys to SSC, a critical concern for components operating in sour environments. Over the years, various modifications have been introduced to this test to enhance its accuracy and reproducibility. This study presents a detailed numerical investigation of the DCB test, adhering to the procedural steps outlined in the ANSI/NACE TM0177 standard to simulate the test. The primary objective is to simulate sulfide stress cracking in the DCB test and analyze the effect of different test parameters on the measured values of $K_{\mathrm{ISSC}}$. The numerical simulations successfully replicate the DCB test steps and accurately capture the evolution of the SIF *K* over time, leading to precise predictions of $K_{\mathrm{ISSC}}$. The results demonstrate a good correlation between the numerical predictions and experimental observations, especially in measuring $K_{\mathrm{ISSC}}$ for various initial applied loads $K_{\mathrm{Iapplied}}$, which correspond to different arm displacements and initial notches.

Additionally, the numerical results accurately capture the relationship between $K_{\mathrm{Iapplied}}$ and $K_{\mathrm{ISSC}}$, consistent with findings from experimental studies using standard DCB specimens. This study also underscores the efficacy of employing an advanced coupled deformation-diffusion phase field modeling approach to simulate the interplay between hydrogen diffusion, material deformation, and cracking in standardized test procedures for sulfide stress cracking, such as DCB testing, which are employed to assess materials in different aqueous test solutions mimicking oilfield environments. Looking forward, this research has significant implications for developing enhanced quality assurance and defect assessment methodologies through refined standardized testing procedures. Furthermore, the study highlights the importance of adopting innovative multiphysics computational modeling methodologies to improve the reliability and longevity of critical infrastructure components, such as pipelines, downhole tubulars, and other ancillary equipment, exposed to harsh environmental conditions.

## CRediT authorship contribution statement

**Alok Negi:** Writing – original draft, Visualization, Methodology, Investigation, Conceptualization. **Mohamed Elkhodbia:** Writing – review & editing. **Imad Barsoum:** Writing – review & editing, Supervision, Resources, Project administration, Funding acquisition, Conceptualization. **Akram AlFantazi:** Supervision, Project administration.

## Declaration of Competing Interest

The authors declare that they have no known competing financial interests or personal relationships that could have appeared to influence the work reported in this paper.

## Data Availability

All data and models used during the study appear in the submitted article.

## Appendix A Finite element implementation

The weak forms of the governing equations Eq. (7) (neglecting the body forces), Eq. (8), and Eq. (17) reads,

(A.1) $$\int_{\Omega }g(\phi )\sigma_{0}:\delta \epsilon dV-\int_{d\Omega }\delta u\cdot t\;dS=0$$

(A.2) $$\int_{\Omega }\left[-2(1-\phi )\delta \phi H_{v}+G_{c}(C)\left(\frac{1}{l}\phi \delta \phi +l\nabla \phi \cdot \nabla \delta \phi \right)\right]dV=0$$

(A.3) $$\int_{\Omega }\left[\delta C\left(\frac{1}{D}\frac{dC}{dt}\right)+\nabla \delta C\nabla C-\nabla \delta C\left(\frac{{\overset{¯}{V}}_{H}C}{RT}\nabla \sigma_{H}\right)\right]dV=-\frac{1}{D}\int_{d\Omega }\delta CpdS$$

These weak forms can be discretized using finite elements as,

(A.4) $$u=N_{u}\;a\;,\;\phi =N_{\phi }\;b\;,\;C=N_{c}\;c$$

where **a**, **b** and **c** are the nodal degrees of freedom; $N_{u}$, $N_{\phi }$ and $N_{c}$ are the corresponding shape function matrices. Similarly, the gradient terms can be discretized as,

(A.5) $$\epsilon =B_{u}\;a\;,\;\nabla \phi =B_{\phi }\;b\;,\;\nabla C=B_{c}\;c$$

where $B_{u}$, $B_{\phi }$ and $B_{c}$ are the gradient operator matrices. After substituting the FE discretization, the residuals for the governing equations of displacement **u**, *ϕ*, and *C* reads,

(A.6) $$R_{u}=g(\phi )\int_{\Omega }{B_{u}}^{T}\sigma_{0}dV-\int_{d\Omega }N_{u}^{T}t\;dS$$

(A.7) $$R_{\phi }=\int_{\Omega }\{-2(1-\phi ){N_{\phi }}^{T}H_{v}+G_{c}(C)\left(\frac{1}{l}{N_{\phi }}^{T}\phi +l{B_{\phi }}^{T}\nabla \phi \right)\}dV$$

(A.8) $$R_{c}=\int_{\Omega }\{{N_{c}}^{T}\left(\frac{1}{D}\frac{dC}{dt}\right)+{B_{c}}^{T}\nabla C-{B_{c}}^{T}\left(\frac{{\overset{¯}{V}}_{H}C}{RT}\nabla \sigma_{H}\right)\}dV+\frac{1}{D}\int_{d\Omega }{N_{c}}^{T}pdS$$

Time parametrization and an incremental-iterative Newton-Raphson solution scheme are used to solve the coupled system of equations. The linearized system of the equations for the mechanical problem reads,

(A.9) $$K_{\mathrm{aa}}\Delta a=-R_{u}$$

(A.10) $$K_{\mathrm{bb}}\Delta b=-R_{\phi }$$

where

(A.11) $$K_{\mathrm{aa}}=g(\phi )\int_{\Omega }{B_{u}}^{T}C_{0}B_{u}dV$$

(A.12) $$K_{\mathrm{bb}}=\int_{\Omega }\{\left(\frac{G_{c}(C)}{l}+2H\right)+G_{c}(C)l{B_{\phi }}^{T}B_{\phi }\}dV$$

Considering the backward Euler difference method for the temporal discretization, the linearized equation for the parabolic mass diffusion reads,

(A.13) $$K_{\mathrm{cc}}c+M\overset{˙}{c}=0$$

where

(A.14) $$K_{\mathrm{cc}}=\int_{\Omega }\{{B_{c}}^{T}B_{c}-{B_{c}}^{T}\left(\frac{{\overset{¯}{V}}_{H}}{RT}\nabla \sigma_{H}\right)N_{c}\}dV$$

(A.15) $$M=\frac{1}{D}\int_{d\Omega }{N_{c}}^{T}N_{c}dS$$

A staggered approach addresses this system of equations, wherein the multi-field problem is subdivided into sub-problems tackled individually through an incremental-iterative scheme. Initially, elastic deformation influences mass diffusion via the stress field. Subsequently, mass transport influences the fracture resistance of a material by accumulating hydrogen in the fracture process zone. Finally, the H-sensitive *ϕ* decreases the strain energy density.
